# A genetically-encoded biosensor for direct detection of perfluorooctanoic acid

**DOI:** 10.1038/s41598-023-41953-1

**Published:** 2023-09-13

**Authors:** Madison M. Mann, Bryan W. Berger

**Affiliations:** 1 Department of Chemical Engineering, University of Virginia, 102 Engineers Way, Charlottesville, VA 22901 USA; 2 Department of Biomedical Engineering, University of Virginia, Charlottesville, USA

**Keywords:** Chemical engineering, Environmental biotechnology

## Abstract

Determination of per- and polyfluoroalkyl substances (PFAS) in drinking water at the low levels set by regulatory officials has been a major focus for sensor developing researchers. However, it is becoming more apparent that detection of these contaminants in soils, foods and consumer products is relevant and necessary at part per billion and even part per million levels. Here, a fluorescent biosensor for the rapid detection of PFOA was engineered based on human liver fatty acid binding protein (hLFABP). By conjugating circularly permuted green fluorescent protein (cp.GFP) to a split hLFABP construct, the biosensor was able to detect perfluorooctanoic acid PFOA in PBS as well as environmental water samples with LODs of 236 and 330 ppb respectively. Furthermore, *E. coli* cells cytosolically expressing the protein-based sensor were demonstrated to quickly detect PFOA, demonstrating feasibility of whole-cell sensing. Overall, this work demonstrates a platform technology utilizing a circularly permuted GFP and split hLFABP conjugate as a label-free optical biosensor for PFOA.

## Introduction

Per and poly-fluoroalkyl substances (PFAS) are a group of man-made chemicals that have become an urgent public health concern. Referred to as “forever chemicals” by news and media outlets, these fluorochemicals are pervasive environmental contaminants without a robust natural degradation pathway^1,2^. Due to their unique chemical composition, PFAS are water and/or oil resistant which makes them advantageous to use in a wide number of commercial applications. First manufactured in the 1940s, PFAS have been used in a variety of industrial and commercial products including fire-fighting foams, synthetic fabrics, medical devices, food packaging, and cosmetic goods^3–6^. PFAS consist of chains of highly fluorinated carbon atoms bound to polar head groups which are typically carboxylic acids, tertiary amines, or sulfide moieties^1,7^. Over 3000 different types of PFAS have been industrially manufactured with two major categories consisting of perfluorocarboxylic acids (PFCAs) and perfluorosulfonic acids (PFSAs)^1^. Unfortunately, their amphiphilic properties confer high solubility under aqueous conditions, causing these chemicals to be a prevalent, mobile and persistent set of environmental contaminants^8–10^.

Despite only having been manufactured for less than a century, most residents of industrialized countries have been exposed to PFAS^11–13^. Among a representative sample of the U.S. population, 95% of human serum analyses yielded a positive result for PFAS^14,15^. With a half-life of several years, and the inability to naturally degrade, PFAS can accumulate in human tissues through long-term exposure, even with a source containing relatively low concentrations^5,16^. While new toxicological effects are continuing to be discovered, long-term exposure to PFAS, specifically medium chain perfluoroalkyl acids like perfluorooctanoic acid (PFOA), have been linked to numerous health problems including increased cholesterol levels^17–19^, various cancers^20–22^, and reproductive issues^23^. Many reviews summarizing toxicity and health information have been published^5,23–26^. Furthermore, biomonitoring studies in a variety of species have shown that upon accumulation, the highest concentrations of PFOA to be found in the blood plasma and liver^27,28^. This has since been further elucidated as PFOA is shown to bind to relevant proteins including liver fatty acid binding protein (LFABP) and serum albumin^29,30^.

With a rise in evidence of PFAS accumulation and toxicity comes a wave of regulatory changes and calls for action that highlight the necessity of quick, relatively easy ways to detect chemicals like PFOA^31,32^. However, this challenge has proven non-trivial given the diversity of the chemicals as well as their limited reactivity and vast concentrations ranges. Currently, standard PFAS detection relies on chromatography techniques coupled with tandem mass spectroscopy. These methods are highly precise with detection limits in the range of 1 ng/L (1 ppt) for aqueous samples (EPA Methods 533, 537, and 537.1)^33–35^. Increasing health concerns and new regulations in response to these concerns have led to development of new PFAS sensors capable of detecting compounds in drinking water. While still constrained to bench level research, the most successful technologies revolve around the use of molecularly imprinted polymers (MIPS) to capture and detect PFAS down to the ppt levels determined in health advisories set by the EPA^32,36–38^. Nonetheless, these methods often require extensive sample preparation, and/or are limited to drinking water samples making PFAS detection impractical for other applications and inaccessible for most communities.

Human exposure to PFAS has been contributed to multiple pathways including ingestion of contaminated water and food including crops grown in contaminated soils and biosolids as well as general dermal adsorption^39^. Therefore, as PFAS contamination is found to be more and more ubiquitous in the environment, the necessity of detection at these higher concentrations and in a vast variety of matrices becomes clearer. In fact, several states in the U.S. report PFOA and PFOS concentrations in the part per million (ppm) in non-drinking water and various soil sources^40,41^. High levels of PFAS pollution are common near manufacturing facilities, sites storing PFAS related waste, and areas utilizing fluorinated AFFFs like airports^42^. In fact, limited analyses of ground water and sediment samples in locations where PFAS containing AFFs were used, have demonstrated that a wide variety of PFAS chemicals can persist in the environment at high concentrations, several decades after release^4,43–46^.

Agricultural lands have become reservoirs for PFAS as these chemicals are emitted directly into the environment or brought in through irrigation waters and soil amendments like treated sewage sludge and soil conditioners^10,47,48^. Therefore, this contamination is reflected in food crops. Studies have shown plants are capable of taking up and accumulating PFAS with preference to medium and short chain chemicals like PFOA and PFOS^49,50^. Specifically, for highly contaminated areas, this has been shown to range from μg/kg to mg/kg dry weight levels in a variety of crops^51–53^, which is orders of magnitude above the limits set for drinking water. With this is mind, there is a clear need for easy and rapid PFAS detection in a multitude of matrices and in a wide range of concentrations with minimal pre-processing. In order to grow necessary food crops and livestock, and allow safe use of outdoor recreation areas, people must be able to easily determine contamination.

Biosensors have often been used for detection of pathogens and contaminates in agricultural products and environmental samples as they offer the advantage of tunability in terms of sensitivity and selectivity as well as the possibility for minimal sample pre-treatment^54–57^. Despite not having been assessed for real world feasibility, several biosensor platforms have been developed for PFAS detection. Some of these technologies are considered “whole cell biosensors”, created around bacteria with engineered biological promoters that induce fluorescent protein expression upon PFAS interaction^58,59^. However, these have yet to be optimized for quick read-outs, often taking 24–48 h. Biosensors utilizing individual binding proteins and antibodies as PFAS receptors rather than whole cell systems, have also been shown to detect PFOA and/or PFOS with various transducers including fiber optics^60,61^. We previously designed an acrylodan based fluorescent sensor for detection of several PFAS in water based on human liver fatty acid binding protein (hLFABP)^62^. While promising, to utilize the robustness and ease of whole-cell biosensors for direct detection in a variety of solid sample formats, it is advantageous to develop a genetically encoded system or protein capable of intrinsic detection of PFAS.

**Figure 1:**
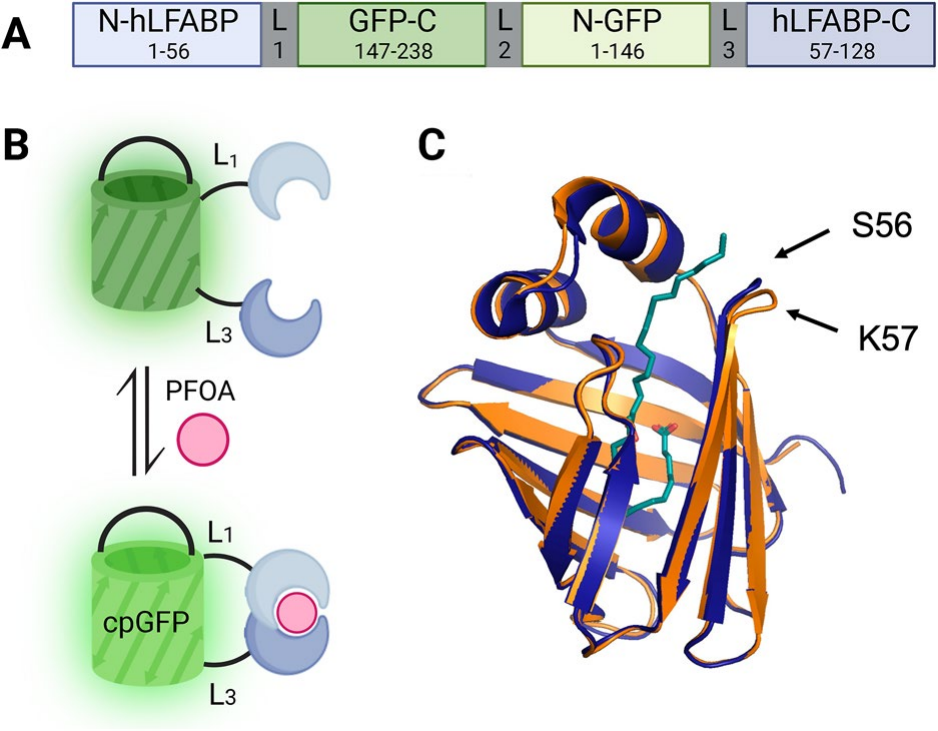
cpGFP.hLFABP construct design overview. **(A)** linear representation of construct with linkers L1 (GSG) L2 (GGTGGS) and L3 (GG), **(B)** schematic of circularly permuted GFP.hLFABP at the protein level depicting binding of PFAS, **(C)** overlay of apo form (orange) and holo form (blue) of wild type hLFABP complexed with palmitic acid (PBD IDs 3STN and 3STK respectively)^77^. Location of receptor splitting was partly based on perturbations at residues 56 and 57. Figure created using Biorender.com.

In this study, we introduce a biosensing scaffold capable of detecting PFOA in aqueous solutions based on hLFABP and utilizing circularly permuted green fluorescent protein (GFP) that can be further optimized for whole cell detection. This fusion protein construct shown in Fig. 1A, B, exhibits increased intrinsic fluorescence upon PFOA binding in vitro with a LOD of 236 ppb, a level well within concentration ranges seen in highly contaminated sites. This is also achieved with minimal protein expression and purification steps and no secondary, post purification modifications. We also demonstrate the feasibility of this construct to be utilized in vivo through cytosolic *E. coli* expression. Our results provide a promising detection platform for use in non-aqueous and heterogeneous media detection as whole cell sensors offer robustness, ease of use, application flexibility as compared to sensors based on purified proteins^63^.

## Materials and methods

### Molecular biology

For DNA maintenance, *E. coli* strain DH5α was used and *E. coli* strain BL21 (DE3) was used for protein expression. Unless otherwise stated, all molecular biology procedures for PCR amplification, plasmid preparation, cell transformation and subcloning were performed according to standard methods supplied by manufacturers. The gene encoding *E. coli* codon optimized human liver fatty acid binding protein (hLFABP) (NCBI 2168) was previously subcloned into pET-28a(+) using *BamHI/XhoI* restriction sites^62^ and the cp.GFP.PPYF gene fragment was amplified from EcMBP165-cpGFP.PPYF.pRSET (Addgene plasmid #33372)^64^. The cp.GFP.PPYF fragment as well as the destination vector, hLFABP-pET28a(+) were amplified using primer sets FragmentCP.GFP and VectorCP.GFP respectively. The insert and linear vector were then simultaneously digested and ligated via Golden Gate assembly utilizing *PaqCI* (New England Biolabs). The ligated cpGFP-hLFABP sequence in pET-28a(+) was verified by Sanger Sequencing (Europhins Genomics).

FragmentCP.GFP_F: *5' TATCACCTGCACTAggcagcggcagctacaacgtcttcat 3'*

FragmentCP.GFP_R: *5' TATCACCTGCACTAacccccgttaaagttgtactccagcttg 3'*

VectorCP.GFP_F: *5'TATCACCTGCACTAgggtaaagtgatccaaaacgaatttaccgttg 3'*

Vector.CP.GFP_R: *5' TATCACCTGCACTAtgccgctgcccgcggtaat 3'.*

### Protein expression and purification

The recombinant protein was produced after transformation into *E. coli* BL21 (DE3). Saturated cell solutions were pelleted by centrifugation (3000*g*), resuspended in fresh LB, and grown at 37 °C to an OD600 of 0.6. Protein expression was then induced via addition of 1mM isopropyl β-d-1-thiogalactopyranoside (IPTG), and cultures transferred to 20 °C for 18 h. Harvested cells were then pelleted by centrifugation (10,000*g*) and resuspended in lysis buffer (50mM Tris–Cl, 100 mM NaCl, 5% v/v glycerol, 1 mM phenylmethylsulfonyl fluoride) before lysis via sonication. The clarified supernatant was then purified by Ni^2+^-affinity chromatography using Chelating Sepharose Fast Flow (Cytiva). The 1 mL column was equilibrated with 50 mM Tris–Cl buffer (pH 8) containing 10 mM imidazole, and protein was separated using a stepwise elution of imidazole up to 500 mM. The fractions collected were analyzed by SDS-PAGE to determine purity. Pure fractions were then pooled and dialyzed against phosphate buffered saline (PBS) pH 7.6. Concentration of all protein samples was measured using a Pierce BCA protein assay kit.

### Spectroscopy

All absorbance and fluorescence measurements for cpGFP.hLFABP were performed using a Synergy Neo2 Hybrid Multi-Mode Microplate Reader (Biotek) at room temperature and under steady state conditions.

### In vitro assays

PFOA binding assays in vitro were performed by titrating PFOA into protein (1 µM final concentration) in either PBS buffer (pH 7.6) or creek water taken from the Dell and Meadow Creek on the grounds of University of Virginia. To determine sensor ability in systems containing other anionic surfactants, the assay was also performed in PBS buffer (pH 7.5) with sodium dodecyl sulfate (SDS) at a final concentration of 1 µM. Samples were allowed to equilibrate for 5 min before fluorescence spectra were recorded over 500–600 nm after excitation at 395 and 485 nm. For more quantitative data, fluorescence intensity endpoint reads at 510 nm after excitation at 485 nm were also collected. The dissociation constant (K_d_) was determined by fitting the fractional change in fluorescence intensity (F) at 510nm and corresponding PFOA concentrations to a one-site binding model using nonlinear regression after correcting for protein in buffer only fluorescence:

1 $$\frac{\Delta \mathrm{F}}{{\mathrm{F}}_{0}}=\frac{\Delta \mathrm{F}}{{\rm F_{0_\mathrm{max}}}}*\frac{[\mathrm{PFOA}]}{({\mathrm{K}}_{\mathrm{d},\mathrm{PFOA}}+\left[\mathrm{PFOA}\right])}$$

### In vivo assays

For in vivo assays, the induced cells were harvested and resuspended in PBS (pH 7.6) to cut down on background media fluorescence. After titration with PFOA, cells were allowed to equilibrate for 30 min at room temperature. After 30 s of orbital shaking, the fluorescence spectra as well as endpoint data were collected as described above. After fluorescence data was normalized by OD600, the fractional change in fluorescence intensity at 510 nm was plotted against PFOA concentration. However, to account for nonspecific interactions, the data were fit to a dose- response model where a half maximal effective concentration (EC50) was obtained:

2 $$\frac{\Delta \mathrm{F}}{{\mathrm{F}}_{0}}= {\frac{\Delta \mathrm{F}}{{\mathrm{F}}_{0_{\rm min}}}}+\frac{{\frac{\Delta \mathrm{F}}{{\mathrm{F}}_{0_{\rm max}}}}-{\frac{\Delta \mathrm{F}}{{\mathrm{F}}_{0_{\rm min}}}}}{{\left(1+{\frac{EC50}{\left[PFOA\right]}}^{HS}\right)}}$$

### Limit of detection calculations

The limits of detection (LOD) were calculated for each non-linear system similarly to existing literature^65^. The base equation (Eq. 3) determines LOD, represented by the deviation in concentration ($${x}_{D})$$, by multiplying the standard deviation of blank samples, ($${s}_{y0})$$, with the coefficient for a Student’s t distribution, $$(t)$$.

3 $${x}_{D}=t*{s}_{y0}$$

While this is often the simplest method for LOD determination, it does not accurately consider response deviation, nor does it measure standard deviation of calibration measurements. For in vitro assays, we see a first order binding dependence of signal on PFOA concentration shown in Eq. (4) after rearrangement and simplification of Eq. (1). Concentration of PFOA is now represented as $(x$) while the change in fluorescence response is now $(y$) with max response as $(B$). Therefore, to properly obtain LOD, we must calculate the contribution of all terms in Eq. (4) in the deviation of $x$*.*

4 $$x=\frac{B*{K}_{d}}{(B-y)}$$

5 $${s}_{x}^{2}={\left(\frac{\partial x}{\partial y}\right)}^{2}{s}_{y}^{2}+{\left(\frac{\partial x}{{\partial K}_{d}}\right)}^{2}{s}_{Kd}^{2}+{\left(\frac{\partial x}{\partial B}\right)}^{2}{s}_{B}^{2}$$

Standard deviation of $$x$$, $${(s}_{x})$$ was calculated based on Eq. (5) with $${(s}_{y})$$, $${(s}_{Kd})$$, and $${(s}_{B})$$ representing error in the calibration curve from the response signal, dissociation constant, and maximum signal respectively. The calculated deviation obtained at a specific point, ($${s}_{y0})$$, from Eq. (5) was substituted into Eq. (3) to calculate LOD. As our system is based on the change in fluorescence, measurement deviation from a low concentration standard was used for $${s}_{y0}$$ rather than blank samples. The final equation (Eq. 6) was then used for calculation of LOD for in vitro assays with the confidence factor t = 3 as it corresponds to the confidence level of 95%.

6 $${x}_{D}=t*{\left[{\left(\frac{{K}_{d}*B}{{\left(B-{y}_{0}\right)}^{2}}\right)}^{2}{s}_{y0}^{2}+{\left(\frac{{-K}_{d}*{y}_{0}}{{\left(B-{y}_{0}\right)}^{2}}\right)}^{2}{s}_{B}^{2}{+\left(\frac{{y}_{0}}{\left(B-{y}_{0}\right)}\right)}^{2}{s}_{Kd}^{2}\right]}^\frac{1}{2}$$

A similar process was used for the LOD calculations for the cell-based assays. However, Eq. (2) was used as the basis instead of Eq. (1) in order to properly model the non-specific effects that come with the complexity of using whole cells instead of purified protein for binding assays.

## Results and discussion

Fluorescent proteins (FPs) contain optical properties that are extremely dependent on the microenvironment surrounding their chromophores^66,67^. This chromophore sensitivity has led to the widespread use of FPs like GFP as sensing tools since small changes to protonation equilibrium are transduced easily. This can be through direct interaction of analytes and chromophores like with FP based pH and ion sensors^68^ or through the addition of a separate binding unit where ligand induced conformation change leads FRET-based activity or even allosteric based fluorescence changes^69–71^. While the addition of ligand-binding receptors to FPs is conducive for binding events that lead to large changes in receptor conformation, the use of circularly permuted GFP has been proven to be capable of transducing binding events for proteins with a wide range of conformational flexibilities^72^. The circular permutation process involves fusing the natural GFP termini which forms new termini that can then be fused to insert a receptor of interest closer to the chromophore^73^.

To create a sensor capable of detecting PFOA, circularly permuted GFP (cpGFP) and human liver fatty acid binding protein were utilized as optical signal and recognition units respectively. The chosen receptor, hLFABP has not only been shown to bind PFOA with moderate affinity^74,75^ but has also been used previously as a scaffold for a PFAS biosensor not suitable for genetic encoding^62^. However, hLFABP is not known to have extensive conformational changes upon binding of endogenous fatty acids^76,77^, and the little structural information of PFOA binding shows only minimal changes in alpha helical composition^30,75^. Therefore, it is imperative that the cpGFP construct is fused near the PFOA binding region of hLFABP without interrupting residues directly responsible for ligand interactions. The residues S56 and K57 in the loop region shown in Fig. 1C were chosen for domain splitting. While located in a flexible region away from residues taking part in electrostatic interactions^74,75^, S56 and K57 are in a region where modest change in structure occurs upon binding of palmitic acid as shown in Fig. 1C as the overlay of the apo and holo form of hLFABP. (PDB 3STN and 3STK respectively)^77^. Furthermore, previous work in our lab has shown insertion of the fluorophore, acrylodan, into a reasonably close residue (F50) of hLFABP is able to probe PFOA binding^62^. Figure 1A also shows a linear map of the construct as well as a graphical overview of the sensor function. As ligand is bound by the split hLFABP, the change in microenvironment around cpGFP’s chromophore will elicit a change in fluorescence. Specifically, this change is seen as an increase in fluorescence after excitation.

**Figure 2:**
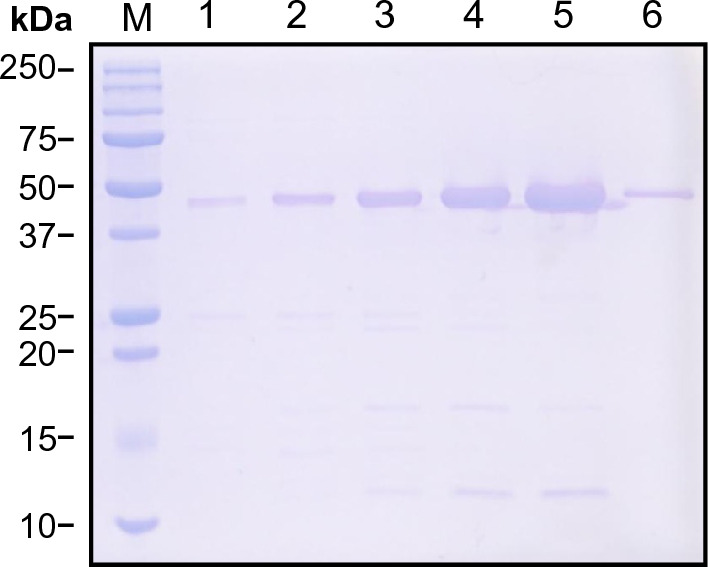
SDS-PAGE analysis of cpGFP.hLFABP purification via Ni–NTA resin. Lane M: protein marker, Lanes 1–6: purification fractions containing increasing concentrations of imidazole (50, 75, 100, 125, 200, and 500mM respectively). cpGFP.hLFABP is shown as a band of approximately 46.6 kDa.

The designed construct was subsequently cloned as described above, expressed in *E. coli* BL21 (DE3), and purified via immobilized metal affinity chromatography. Purified fractions containing the single band of cpGFP.hLFABP at ~ 46.5 kDa (Fig. 2) were then dialyzed and used for in vitro analysis.

**Figure 3:**
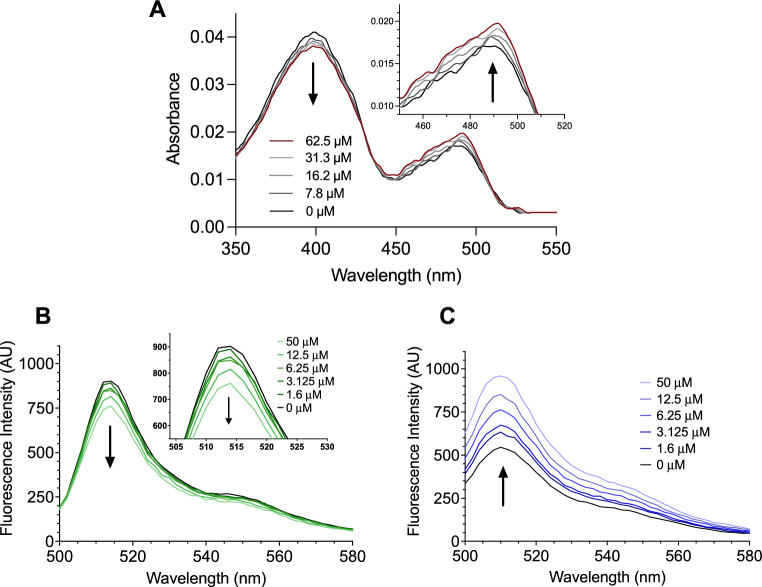
**(A)** Representative absorption spectra for cpGFP.hLFABP (2 μM). Spectral changes upon increased PFOA addition show a decreased and increased trend in intensity at 395 nm and 485 nm respectively. **(B,C)** Representative fluorescence emission spectra for cpGFP.hLFABP (1 μM) after excitation at 395 nm **(B)** and 485 nm **(C)**. Spectral changes upon PFOA titration shows a decreased and increased overall intensity for excitation at 395 nm and 485 nm respectively. All curves are smoothed for qualitative analysis.

Enhanced green fluorescent protein (EGFP), the original basis of this cpGFP signal unit, as well as our cpGFP.hLFABP fusion have absorbance peaks (Fig. 3A) corresponding with the protonated and deprotonated forms at ~ 395 nm and ~ 490 nm respectively^78^. Upon titration of PFOA, the absorbance at 395nm increased and decreased at 485 nm. This indicates shifts in the equilibrium state of the chromophore from protonated to deprotonated states upon binding of PFOA to the split hLFABP.

Fluorescence response at these two wavelengths was assessed upon addition of PFOA. Figure 3B, C show that while emission spectra changes occur at both wavelengths, excitation at 485 nm exhibits a much more exaggerated response in overall fluorescence intensity change.

**Figure 4:**
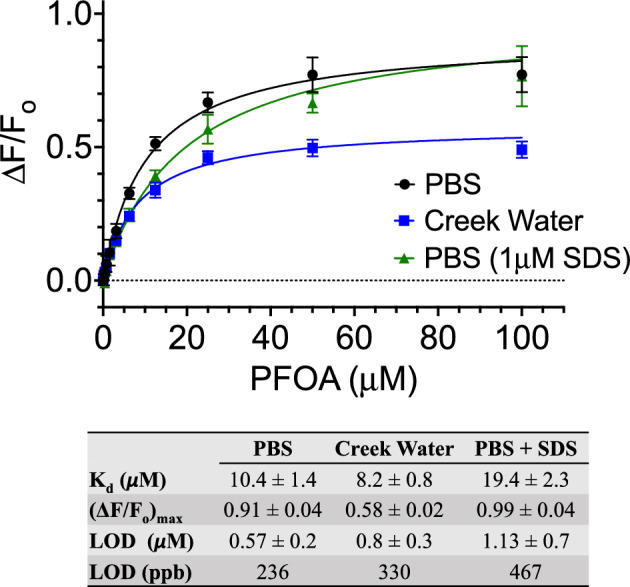
Binding of PFOA to cpGFP.hLFABP (1 µM) after titration in PBS (black circles), creek water (blue squares), and PBS containing 1µM SDS (green triangles). Data are fitted to a one site binding model with the fractional occupancy represented as the fractional change in fluorescence intensity at 510 nm after excitation at 485nm. The represented points are mean values ± SEM with n = 3. Bottom table displays calculated model constants and limits of detection in all systems.

Therefore, to quantify binding of PFOA to cpGFP.hLFABP, endpoint intensity at 510 nm after excitation at 485 nm was used. Figure 4 shows binding of PFOA to cpGFP.hLFABP in PBS (pH 7.6) as a fractional change in fluorescence. This data is shown fitted to a one site binding model based on the best fit to experimental data. This is consistent with studies suggesting PFOA binds in a 1:1 stoichiometry to WT hLFABP despite cpGFP.hLFABP utilizing a split hLFABP domain^30,62,75^. The calculated dissociation constant K_d_ was determined to be 11.9 ± 1.6 μM which is also consistent with previous studies characterizing binding of PFOA to WT hLFABP and variants^30,62,75,79^.

To assess feasibility and selectivity of the sensor in more realistic application systems, these experiments were also done with spiked water samples taken from the Dell and Meadow Creek on the grounds of University of Virginia as well as in buffer containing the anionic surfactant, sodium dodecyl sulfate (SDS), as a competitor for binding (Fig. 4**)**. While K_d_ values obtained in all buffer systems were similar as shown in Fig. 4, the maximum response, and limits of detection (LODs) varied. While maximum response, (ΔF/F_o_)_max_, is reduced due to outside allosteric interactions in creek water-based assays, the overall binding affinity and limits of detection remain comparable to PBS based data with LODs in the hundreds of ppb. While this limit is pushed toward the part per million for buffer containing SDS, the ability to detect PFOA at this level in a system with a known hLFABP binder is promising for application of samples that contain other anionic surfactant co-contaminants.

### In vivo

One of the most valuable aspects of genetically encoded sensors is the ability to be used in vivo*.* Specifically, in environmental detection, utilizing whole bacterial cells introduces a robustness to a sensor as compared to proteins alone in terms of tolerance to physical changes like pH and temperature as cell membranes act as a barrier from harsh environmental conditions^80^. Furthermore, whole cell-based systems are usually more amenable to immobilization-based implementation than proteins^81^ and offer advantages such as portability and the possibility of detection in complex matrices with minimal sample preparation^82^.

**Figure 5:**
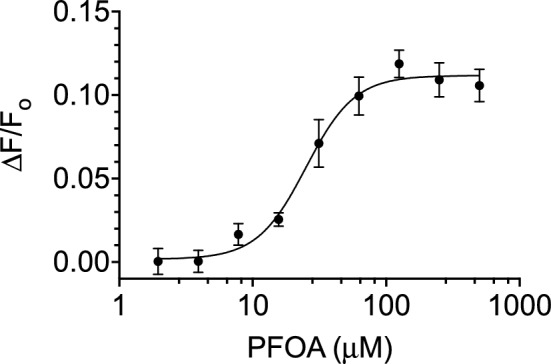
Fluorescence intensity change at 510nm after excitation at 485nm of *E. coli* expressing cpGFP.hLFABP upon PFOA titration. Data are normalized by cell concentration (OD600) and fitted to a log-dose response model. The represented points are mean values ± SEM with n = 3.

Therefore, as a first pass at feasibility of whole cell detection, *E. coli* cells expressing cpGFP.hLFABP cytosolically were incubated with PFOA, and changes in fluorescence monitored. The increase in fluorescence upon PFOA titration is shown in Fig. 5**.** To account for complications the bacterial interactions bring, the data was fit to a log-dose response model rather than a one site binding model. While only eliciting a maximal response of around 10%, the sensor expressing cells are capable of detecting PFOA in PBS (pH 7.6) with a LOD of 2.4 ppm.

Overall, in this study we demonstrate the design and initial applicability of an intrinsic fluorescent PFOA sensor based on conjugation of cpGFP to a split hLFABP construct. The purified fusion protein exhibited dose-dependent changes in absorption spectra as well as fluorescence intensity that demonstrate saturation behavior. This shows that despite small changes in structural conformation, splitting hLFABP allows PFOA induced changes large enough for transduction by cpGFP without detrimental changes to binding as compared to wild type hLFABP.

Direct detection of PFOA was achieved in buffer as well spiked water samples with LODs within relative environmental concentration ranges without sample pre-treatment. We also demonstrate the feasibility of utilizing this construct in whole bacterial cells through cytosolic expression to detect PFOA. This is extremely promising as minimal optimization was done in terms of individual GFP and hLFABP moieties. As has been shown prior, just optimizing the linkers between cpGFP and receptor proteins can improve optical signal by more than 200%^64^.

As further evidence continues to elucidate the wide-reaching contamination of PFAS chemicals, the need for detection strategies grows. Specifically, detection in a wide variety of sample types and concentrations are necessary. As mentioned above, biosolids and irrigation waters have become reservoirs as well as transporters of PFAS to crops, livestock, and subsequently, people. In the last 10 years, PFAS have been found at detectable levels not only in food crops but also livestock, including milk from dairies with groundwater and hay contamination^83^. Therefore, to address the need for easy detection in all sample types, our lab looks towards utilizing synthetic biology. At the bench scale, biosensor feasibility studies are usually conducting in liquid culture. However, genetically encoded biosensors have the capability for a multitude of applications including immobilization for the design of test strips, and microfluidic devices^63,81^. We also know that with biological tool kits, researchers have the capability to modify proteins and enzymes to enhance sensitivity and selectivity drastically. We hope this work lays a foundation for biological detection of PFOA as well as other PFAS molecules including sulfonated chemicals like PFOS as well as long-chain fluorotelomers. Furthermore, genetically encoded biosensors can be designed with multiple functions. By introducing new proteins and enzymes or creating genetic circuits, whole cell sensors seem to have unlimited capabilities, including pre-treatment simplification. Therefore, by demonstrating cpGFP.hLFABP as a promising platform for intrinsic fluorescent-based detection, we hope to enable further synthetic biology-based approaches for PFAS detection.

## Data Availability

The data sets generated during and/or analyzed during the current study are available from the corresponding author on reasonable request.
